# A 16-year bicentric retrospective analysis of ovarian tissue cryopreservation in pediatric units: indications, results, and outcome

**DOI:** 10.3389/fendo.2023.1158405

**Published:** 2023-08-31

**Authors:** Marine Grellet-Grün, Béatrice Delepine, Pauline Le Van Quyen, Gerlinde Avérous, Anne Durlach, Cécile Greze, Laetitia Ladureau-Fritsch, Isabelle Lichtblau, Anne-Sophie Canepa, Antoine Liné, Catherine Paillard, Claire Pluchart, Olivier Pirrello, Catherine Rongieres, Ghassan Harika, François Becmeur, Marius Teletin

**Affiliations:** ^1^ Department of Reproductive Biology – Centre d’Etude et de Conservation des Oeufs et du Sperme Humain (CECOS), Centre Hospitalier Universitaire de Reims, Reims, France; ^2^ Department of Pathology, Hôpital de Hautepierre, Strasbourg, France; ^3^ Department of Pathology, Centre Hospitalier Universitaire de Reims, Reims, France; ^4^ Department of Reproductive Biology – Centre d’Etude et de Conservation des Oeufs et du Sperme Humain (CECOS), Centre Médico-chirurgical Obstétrique, Schiltigheim-Strasbourg, France; ^5^ Department of Pediatric Surgery, Centre Hospitalier Universitaire de Reims, Reims, France; ^6^ Department of Pediatric Onco-Hematology, Hôpital de Hautepierre, Strasbourg, France; ^7^ Department of Pediatric Onco-Hematology, Centre Hospitalier Universitaire de Reims, Reims, France; ^8^ Department of Gynecology-Obstetric, Centre Médico-Chirurgical Obstétrique, Schiltigheim–Strasbourg, France; ^9^ Department of Gynecology-Obstetric, Centre Hospitalier Universitaire de Reims, Reims, France; ^10^ Department of Pediatric Surgery, Hôpital de Hautepierre, Strasbourg, France; ^11^ Institut de Génétique et de Biologie Moléculaire et Cellulaire (IGBMC), Centre National de la Recherche Scientifique (CNRS UMR7104), Institut National de la Sante et de la Recherche Médicale (INSERM U1258), Université de Strasbourg (UNISTRA), Illkirch Graffenstaden, France

**Keywords:** fertility preservation, children cancer, ovarian tissue cryopreservation, gonadotoxic treatment, follow up

## Abstract

**Background:**

Cancer treatments of the last decades improve the survival rate of children and adolescents. However, chemo- and radiotherapy result in gonadal damage, leading to acute ovarian failure and sterility. The preservation of fertility is now an integral part of care of children requiring gonadotoxic treatments. Ovarian tissue cryopreservation (OTC) is an effective fertility preservation option that allows long-term storage of primordial follicles, subsequent transplantation, and restoration of endocrine function and fertility. The efficacy of this technique is well-demonstrated in adults but the data are scarce for pediatric patients. Currently, OTC represents the only possibility of preserving the potential fertility in prepubertal girls.

**Procedure:**

This is a retrospective study of OTC practice of two French centers from January 2004 to May 2020. A total of 72 patients from pediatric units underwent cryopreservation of ovarian tissue before gonadotoxic therapy for malignant or non-malignant diseases. The ovarian cortex was cut into fragments and the number of follicles per square millimeter was evaluated histologically. The long-term follow-up includes survival rate and hormonal and fertility status.

**Results:**

The mean age of patients at OTC was 9.3 years [0.2–17] and 29.2% were postpubertal; 51 had malignant diseases and 21 had non-malignant diseases. The most frequent diagnoses included acute leukemia, hemoglobinopathies, and neuroblastoma. Indication for OTC was stem cell transplantation for 81.9% (*n* = 59) of the patients. A third of each ovary was collected for 62.5% (*n* = 45) of the patients, a whole ovary for 33.3% (*n* = 24) of the patients, and a third of one ovary for 4.2% (*n* = 3) of the patients. An average of 17 fragments [5–35] per patient was cryoconserved. A correlation was found between the age of the patients and the number of fragments (*p* < 0.001). More fragments were obtained from partial bilateral harvesting than from whole ovary harvesting (*p* < 0.05). Histological analysis of ovarian tissue showed a median of 6.0 primordial follicles/mm^2^ [0.0–106.5] and no malignant cells were identified. A negative correlation was found between age and follicular density (*p* < 0.001). Median post-harvest follow-up was 92 months [1–188]. A total of 15 girls had died, 11 were still under treatment for their pathology, and 46 were in complete remission. Of all patients, 29 (40.2%) were subjected to a hormonal status evaluation and 26 were diagnosed with premature ovarian insufficiency (POI) (*p* < 0.001). One patient had undergone thawed ovarian tissue transplantation.

**Conclusion:**

OTC should be proposed to all girls with high risk of developing POI following gonadotoxic therapies in order to give them the possibility of fertility and endocrine restoration.

## Introduction

Cancer treatments of the last decades improve the survival rate of children and adolescents; however, chemo- and radiotherapy result in gonadal damage, leading to acute ovarian failure and sterility. The current 5-year survival rate for girls treated for cancer is more than 80%, which will lead to an increase in adult women survivors after a malignancy (1).

The preservation of fertility is now an integral part of care of children and adult female requiring gonadotoxic treatments (2). Among different methods of fertility preservation, ovarian tissue cryopreservation (OTC) followed by ovarian tissue transplantation (OTT) has proved to be a valid strategy to preserve the endocrine and reproductive functions in women with a high risk of premature ovarian failure (3–5). OTC currently represents the only method feasible in prepubertal girls (6, 7). OTC is an effective fertility preservation option not only for oncological patients but also for those with non-malignant diseases requiring bone marrow transplantation (e.g., autoimmune diseases and B-thalassemia) (8).

Since 1996 when the first OTC was proposed (9), several series of patients have been reported. However, most of these studies focus on adult patients, and data concerning pediatric populations remain scarce (6, 10–22). The absence of international registries and the lack of reported results in many centers yield a yet uncomplete picture of the outcome after OTC.

The purpose of this article is to report 16 years of experience in OTC in patients up to the age of 18 years in two different pediatric units concerning patient’s characteristic and follow- up of the cohort.

## Materials and methods

### Patients

This is a retrospective study of OTC practice of two French pediatric centers from January 2004 to May 2020. A total of 72 patients from pediatric units underwent cryopreservation of ovarian tissue before highly gonadotoxic therapy for malignant or benign diseases. The indication of OTC was established when the treatment included the following: conditioning for autologous or allogeneic hematologic stem cell transplantation, high‐dose chemotherapy, total body or pelvic irradiation, or ovariectomy.

### Ovarian tissue retrieval

Ovarian tissue retrieval, performed mainly by laparoscopy or minilaparotomy, consisted of either unilateral ovariectomy or bilateral partial ovariectomy at about one-third of the organ. The ovarian tissue was transferred to the laboratory in less than 1 h in a transport medium Leibovitz 15 (Life Technologies, Cergy Pontoise, France) on ice.

### Ovarian tissue cryopreservation

The ovarian cortex was isolated from the medulla and then cut into fragments of 3 × 5 mm with a thickness of 1–2 mm. Each fragment was placed in a straw (CryoBioSystem) or cryotube (Nunc, Poly Labo, Strasbourg France) and then in high-security tubes (CryoBioSystem) containing 1 ml of freezing solution composed of the transport medium supplemented with 1.5 mol/L dimethylsulfoxide (Wak‐Chemie Medical GmbH, Steinbach, Germany) and 0.1 mol/L sucrose, as cryoprotectant agents and 4 mg/ml HSA (Vitrolife, Göteborg, Sweden) or 10% decomplemented patient serum.

After a 30‐min equilibration in freezing medium for 30 min on ice on a shaking plate, the tubes or straws were placed in a programmable freezer, and the flow temperature was +4°C. Briefly, the temperature drop was initially from 2°C/min to −9°C. After manual seeding, the temperature drop was resumed at a rate of 0.3°C/min to −40°C. The temperature was then dropped from 10°C/min to −140°C. The tubes or straws were finally immersed and stored at the temperature of liquid nitrogen.

A fragment of ovarian cortex was fixed in formalin and processed for histological analysis to evaluate the number of follicles per square millimeter and the presence/absence of malignant ovarian micro metastasis.

### Studied parameters

Several characteristics including age, malignant or non-malignant disease and treatment, and histology parameters of ovarian cortex were extracted from the patients’ medical records. Follow-up data and request for OTT or alternative assisted reproduction technique during follow up were also reviewed. The long-term follow-up included survival rate and hormonal and fertility status.

## Results

### OTC activity

Between January 2004 and May 2020, 72 patients (22 in Reims and 50 in Strasbourg) underwent cryopreservation of ovarian tissue before receiving a highly gonadotoxic treatment. Pediatric patients were referred from two university hospitals (Strasbourg University Hospital and Reims University Hospital).

### Patient age

**Figure 1:**
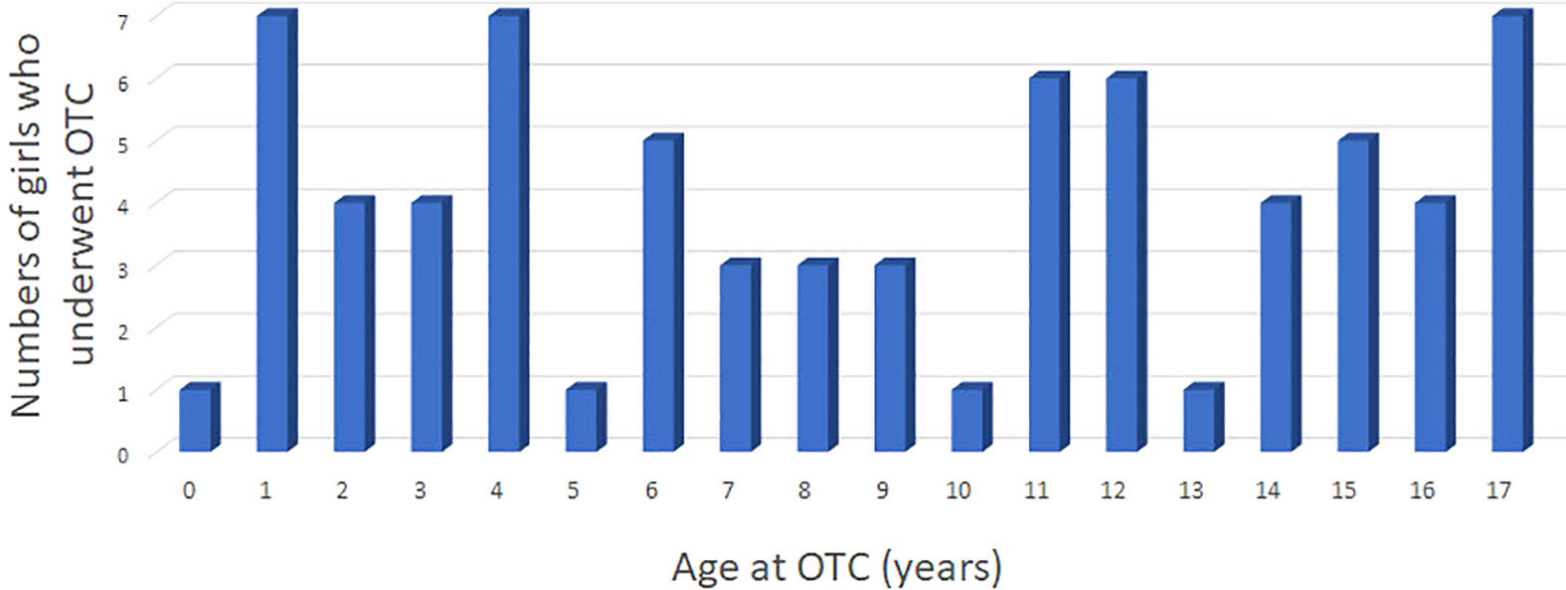
Patient series of 72 girls and adolescents younger than 18 years of age who underwent ovarian tissue cryopreservation between January 2004 to May 2020; blue bars (Strasbourg and Reims).

At the time of OTC, the mean age of 72 patients was 9.3 years [0.2–17] (**Figure 1**). The youngest patient undergoing OTC in the cohort was 2 months old (**Figure 1**). Out of the 72 patients, 51 were prepubertal and 21 were postpubertal. At the time of the study, the patients’ ages ranged from 2 to 33 years, with a mean of 15.4 years. Out of 72 patients, 14 were older than 18 years (19%), and only 5 were older than 23 years (7%). Forty-seven patients were prepubertal at the time of OTC, and 22 were postpubertal, while no information was found for 3 patients regarding hormonal status.

### Diagnosis

Of the 72 patients, 51 had malignant diseases (70.8%) and 21 had non-malignant diseases (29.1%) (**Table 1**). The most frequent diagnoses in this cohort were acute leukemia, hemoglobinopathies, and neuroblastoma. In the group of patients with malignant diseases, 25 had a hematological malignancy (34.7%) with acute lymphoblastic leukemia in 13.9% of cases (*n* = 10) and 26 had a solid tumor (36%) with neuroblastoma in 13.9% of cases (*n* = 10). Hemoglobinopathies were the most common benign disease in the cohort accounting for 21% of cases (*n* = 15). Stem cell transplantation was performed, post-OTC, in 80.6% (*n* = 58) of the patients.

**Table 1:** Diseases of the 72 girls who underwent OTC.

			*N*	%
Malignant diseases (*n* = 51; 71%)	Malignant hematological diseases (*n* = 25; 35%)	Acute lymphoblastic leukemia	10	13.9
		Hodgkin lymphoma	7	9.7
		Acute myeloblastic leukemia	6	8.3
		Non-Hodgkin lymphoma	1	1.5
		Myelodysplastic syndrome	1	1.4
	Solid malignant tumors (*n* = 26; 36%)	Neuroblastoma	10	13.9
		Ewing sarcoma	4	5.6
		Medulloblastoma	3	4.2
		Nephroblastoma	2	2.8
		Osteosarcoma	1	1.4
		Hypothalamic germinoma	1	1.4
		Uterine rhabdomyosarcoma	1	1.4
		Peripheral nerve sheath sarcoma	1	1.4
		Sacrococcygeal immature teratoma	1	1.4
		Thymoma	1	1.4
		Desmoplastic small round cell tumor	1	1.4
Non-malignant diseases (*n* = 21; 29%)	Hemoglobinopathies (*n* = 15; 21%)	Sickle cell disease	10	13.9
		Beta-thalassemia major	5	6.9
	Other non-malignant diseases (*n* = 6; 8%)	Lymphohistiocytosis	2	2.8
		Mature teratoma of the ovary	2	2.8
		Idiopathic medullary aplasia	1	1.4
		Systemic lupus erythematosus	1	1.4

### Histological analysis

**Figure 2:**
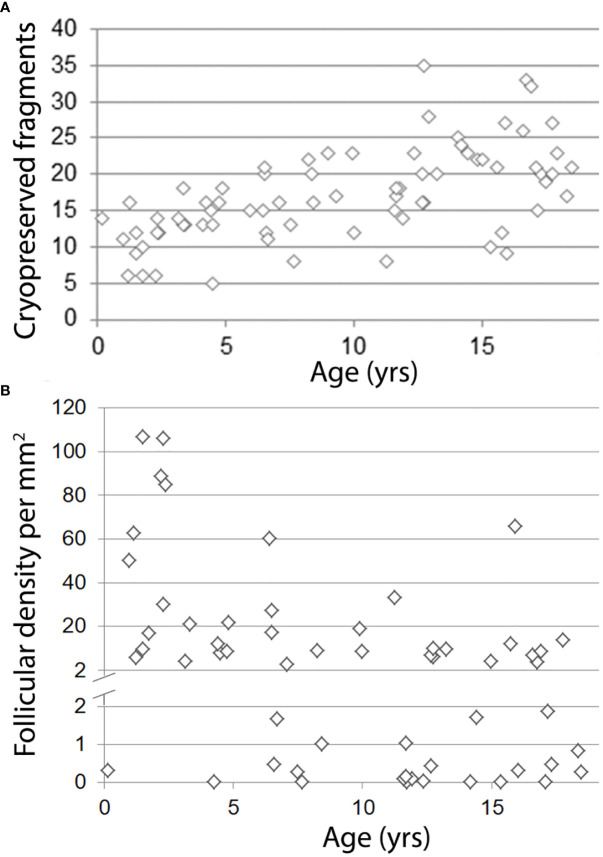
Correlation between age and number of cryopreserved fragments **(A)** or density of primordial follicles **(B)**. A: *R*² = 0.36; *p* < 0.001, B: *R*² = 0.21; *p* < 0.001.

An average of 17 fragments [5–35] per patient was cryoconserved. A correlation was found between age and the number of fragments (**Figure 2A**). More fragments were obtained from partial bilateral ovariectomy than from total unilateral ovariectomy (*p* < 0.05). Histological analysis of ovarian tissue showed a median of 6.6 primordial follicles/mm² [0.0–106.5] and no malignant cells were identified. A negative correlation was found between age and follicular density (**Figure 2B**). Mean follicular density was higher (35.27/mm²) in very young patients (0–4 years old) compared to older patients (5–10, 11–14, and 15–18 years old, respectively) (12.2/mm², 4.9/mm², and 9.7/mm², respectively).

### Patient follow-up/Mortality

Median post-harvest follow-up was 92 months [1–188]. During the follow-up period, 15 patients (8 in Reims and 7 in Strasbourg) died (20.8%). The group of patients with the highest death rate had malignant solid tumors (7 patients, 26.9%), followed by malignant hemopathies (*n* = 5, 20.0%). In the non-malignant group, 14.3% (*n* = 3) of the patients with immunodeficiency died. A total of 11 patients were still treated for their pathology (15%) and 46 were in complete remission (64%). Of all patients, 29 were subjected to a hormonal status evaluation and 26 were diagnosed with premature ovarian insufficiency (POI) (oligo/amenorrhoea for at least 4 months and FSH in the menopausal range levels >40 IU/L on two occasions > 6 weeks apart) (*p* < 0.001). Among the prepubertal girls at the time of OTC, 15 remained prepubertal at the time of the study (mean age = 7.6 years), 10 died, 18 needed hormone replacement therapy (HRT), and 2 patients experienced spontaneous puberty. In the postpubertal group at the time of OTC, out of 22 patients, 5 died, and for 6 patients, no information was found regarding hormonal status. Among the remaining 11 postpubertal patients, 10 were under HRT and 1 recovered and had a normal hormonal status.

### Requests for grafting of cryopreserved ovarian tissue

One patient returned to request the transplantation of cryopreserved ovarian tissue. At the time of ovarian storage, the patient was 17 years old and had been diagnosed with stage II Hodgkin’s disease. She immediately started standard chemotherapy, which consisted of six cycles of doxorubicin hydrochloride (Adriamycin), bleomycin sulfate, vinblastine sulfate, and dacarbazine (ABVD). Four weeks later, she underwent a unilateral laparoscopic oophorectomy, and the obtained tissue was divided into 20 fragments. Histological analysis revealed a follicular density of 13.8 follicles per square millimeter and no signs of malignancy.

Subsequently, the patient underwent autologous hematopoietic stem cell transplantation (HSCT). The consolidation treatment included three cycles of Mesna, Ifosfamide, Mitoxantrone, and Etoposide (MINE), and the conditioning treatment before HSCT included Carmustine, Etoposide, Cytarabine, and Melphalan (BEAM). Three months later, she received a new conditioning treatment consisting of cytarabine and melphalan, followed by 12 Gray total body irradiation (TBI) and a second autologous HSCT. Four months later, she received 20 Gray specifically targeted at the cervical region.

The patient was initiated on hormonal replacement therapy (HRT) and continued to undergo regular gynecologic and hematologic follow-up, showing no signs of disease recurrence. OTT was performed 14 years later when she was 31 years old, although the grafting of nine fragments of ovarian cortex has not yet resulted in a pregnancy. Another patient gave birth through egg donation without undergoing OTT. This patient declined transplantation to avoid another surgical procedure.

## Discussion

OTC is still considered an experimental procedure in most countries (3). However, in the last decade, OTC has become the standard care for fertility preservation in both pre- and postpubertal female patients in several countries, including the USA, Israel, Denmark, Norway, and France (23). Additionally, for prepubertal girls, OTC represents the only option for fertility preservation.

Currently, both OTC and OTT procedures are considered safe for adults and children (10). Many young patients who have undergone OTC are still at a young age and have not yet reached a stage where they intend to use the transplantation of thawed ovarian tissue. In our study, only one patient who had undergone OTC during infancy requested OTT. Despite the recovery of hormonal function, pregnancy has not been achieved after three attempts of *in vitro* fertilization. In Strasbourg University Hospitals, four adult patients have undergone OTT, resulting in two pregnancies [one reported in (24) and one not yet reported].

The results of both university hospitals are quite similar in several aspects of OTC, except for the proportion of malignancies versus non-malignant conditions, the type of ovariectomy (partial bilateral in Strasbourg versus total unilateral in Reims), and the death rate. Non-malignant conditions, such as hemoglobinopathies, accounted for 29% of the total cohort, which is higher compared to several reports (e.g., 16% to 25%) (6, 15, 25–27). This higher proportion of non-malignant conditions in Strasbourg (38%) is due to the collaboration with units of hematopoietic stem cell transplantation (HSCT).

The mean age of our population is 9.3 years, which is lower compared to recent reports where the range is between 11 and 13 years (15, 20, 27, 28). In some recently reported pediatric cohorts, the mean age was even lower, ranging from 6.9 to 7.5 years, but these cohorts included only patients under 14 (28) or 15 years (6), respectively. At the time of the study, the patients’ ages ranged from 2 to 33 years, with a mean of 15.4 years. Out of 72 patients, 14 were older than 18 years (19%), and only 5 were older than 23 years (7%). Forty-seven patients were prepubertal at the time of OTC, and 22 were postpubertal, while no information was found for 3 patients regarding hormonal status. Among the prepubertal girls at the time of OTC, 15 remained prepubertal at the time of the study (mean age = 7.6 years), 10 died, 18 needed hormone replacement therapy (HRT), and 2 patients experienced spontaneous puberty. In the postpubertal group at the time of OTC, out of 22 patients, 5 died, and for 6 patients, no information was found regarding hormonal status. Among the remaining 11 postpubertal patients, 10 were under HRT and 1 recovered and had a normal hormonal status.

The size of ovarian tissue fragments is an important parameter for OTC, as smaller sizes have an adverse effect on the graft follicle pool after OTT due to increased activation and loss, known as the “burn-out” effect (29, 30). In our case, the fragments ranged from 3 to 5 mm in size with a thickness of 1 to 2 mm, which is comparable to other studies in children (6) but smaller than reported in other studies (up to 20–30 mm) [reviewed in (31)]. Follicular density is also an important parameter that influences the duration and function of the grafted fragments (32, 33). It is estimated that the majority of primordial follicles are lost during the procedure, particularly after grafting (34). In our study, follicular density, as expected, was highest in younger patients (0–5 years old). The pediatric population in our study represents 38.5% of the total OTC cases, which is consistent with other larger studies (i.e., 40.5%) (6). No complications related to OTC were noted in our cohort.

## Conclusion

The evolving landscape of pediatric clinical oncology demands ongoing advancements, driven by improved survival rates, which create an ethical obligation for clinicians to provide fertility preservation options. When young patients necessitate highly gonadotoxic chemotherapy or radiotherapy, OTC emerges as the sole viable technique to consider. Our findings contribute to the existing understanding of OTC in the pediatric population, and further follow-up studies will offer additional insights into the long-term outcomes of this approach for very young patients. To minimize the risk of POI resulting from gonadotoxic therapies, it is recommended that OTC be proposed to all high-risk girls and young women, but only after ensuring that parents and patients have been fully informed about the procedure’s risk/benefit ratio.

## Data availability statement

The original contributions presented in the study are included in the article/supplementary material. Further inquiries can be directed to the corresponding author.

## Ethics statement

Ethical review and approval was not required for the study on human participants in accordance with the local legislation and institutional requirements. Written informed consent from the participants’ legal guardian/next of kin was not required to participate in this study in accordance with the national legislation and the institutional requirements. This study was exempt from institutional review board approval according to French Law No. 2004-800 of 6 August 2004 on Bioethics whereby it is stated that OTC is part of patient care.

## Author contributions

MG-G, BD, and MT planned the study. FB, AL, and GH performed ovarian tissue retrieval. MT, LL-F, CG, A-SC, IL, and BD cryopreserved ovarian tissue. PQ, GA, and AD performed histological analysis. MG-G, MT, and BD analyzed and collected data, and drafted the paper. CPa and CPl addressed patients for CTO. CR, OP, and GH performed gynecologic and obstetrics follow-up of the patients. All authors contributed to the article and approved the submitted version.
